# Research progress on the mechanistic impact of single-nucleotide polymorphisms and dietary pattern interactions on overweight and obesity in Chinese adults: a narrative review

**DOI:** 10.3389/fnut.2025.1603038

**Published:** 2025-11-18

**Authors:** Weiling Yang, Hong Xu, Chenhao Xu, Keyi Cao, Yinan Pan, Renjie Gu, Qi Zhu, Jing Xiao

**Affiliations:** 1 Institute for Applied Research in Public Health, School of Public Health, Nantong University, Nantong, Jiangsu, China; 2 Department of Chronic Non-Communicable Diseases Prevention and Control, Jiangsu Provincial Center for Disease Control and Prevention, Nanjing, Jiangsu, China; 3 Medical Department of Xinglin College, Nantong University, Nantong, Jiangsu, China

**Keywords:** SNPs, nutrition, obesity, Chinese adults, gene-diet interactions

## Abstract

Obesity is a major global public health challenge caused by a complex interplay of genetic predisposition, environmental factors (notably dietary transitions), and their interactions. In 2025, China's National Health Commission (NHC) launched the “Year of Weight Management” initiative during its key policy-setting sessions, generating substantial public and scientific attention. Despite extensive research on obesity, a comprehensive analysis integrating gene–diet interactions with sex differences specifically in Chinese adults remains lacking. This review synthesizes recent advances in understanding obesity-related gene polymorphisms, dietary influences, and gene–diet interactions in Chinese adults, with particular emphasis on multigene synergistic effects. Our analysis demonstrates that such genetic synergism shows potential for predicting obesity intervention outcomes, while gene–diet interactions substantially contribute to obesity heterogeneity. Furthermore, maintaining a balanced dietary structure is particularly crucial for specific genotypes, with sex differences exerting distinct moderating effects. By transcending conventional single-factor analytical frameworks and incorporating population-specific genetic and dietary backgrounds, this study proposes that future research should prioritize developing a three-dimensional gene–diet–metabolic phenotype model (an integrative framework considering genotype, dietary intake, and dynamic metabolic outcomes) to identify critical windows and mechanisms of metabolic dysregulation. This approach holds substantial promise for informing personalized precision strategies for obesity prevention and control.

## 1 Introduction

Obesity is a chronic and progressive disease that elevates the risk for numerous comorbidities, such as cardiovascular disease, diabetes, other metabolic disorders, depression, and breast cancer (1–3). As such, it represents one of the most pressing public health challenges globally (4). This global concern is mirrored in China, where national survey data reveal that over half of the adult population is now overweight or obese (5), signaling a rapid rise in obesity prevalence. Against this backdrop, the topic of “weight loss for all” gained prominence as a key agenda item during the two sessions of the Healthy China Initiative, highlighting the government's focused commitment to advancing weight management and chronic disease prevention.

The shift from a traditional lifestyle to a modern, obesogenic environment—characterized by pervasive sedentary behaviors, reduced physical activity, and excessive energy intake—is widely recognized as a principal driver of the escalating obesity epidemic in China (6). Within this context, dietary transitions toward increased consumption of cooking oils, animal-source foods, sugar-sweetened products, and energy-dense snacks and fried items have been especially influential (7, 8). It is important to note, however, that while these dietary shifts have collectively driven population-level obesity trends, individual susceptibility to weight gain in response to such an environment varies substantially (9, 10), likely due to genetic susceptibility. Genome-wide association studies (GWAS) have successfully identified multiple obesity susceptibility loci while also elucidating their potential biological mechanisms (11–13). Nevertheless, the collective contribution of these identified single-nucleotide polymorphisms (SNPs) explains less than 5% of the variation in body mass index (BMI)—a figure strikingly lower than the estimated heritability of 40%−70% (14–16). This substantial gap underscores the value of consolidating existing findings to facilitate the discovery of additional loci, potentially through the investigation of gene–gene associations. Recent evidence indicates that gene–environment interactions (GxE) represent a key factor accounting for obesity heterogeneity, providing novel insights into obesity development that cannot be fully explained by genetic susceptibility or dietary intake independently (11, 17).

**Figure 1:**
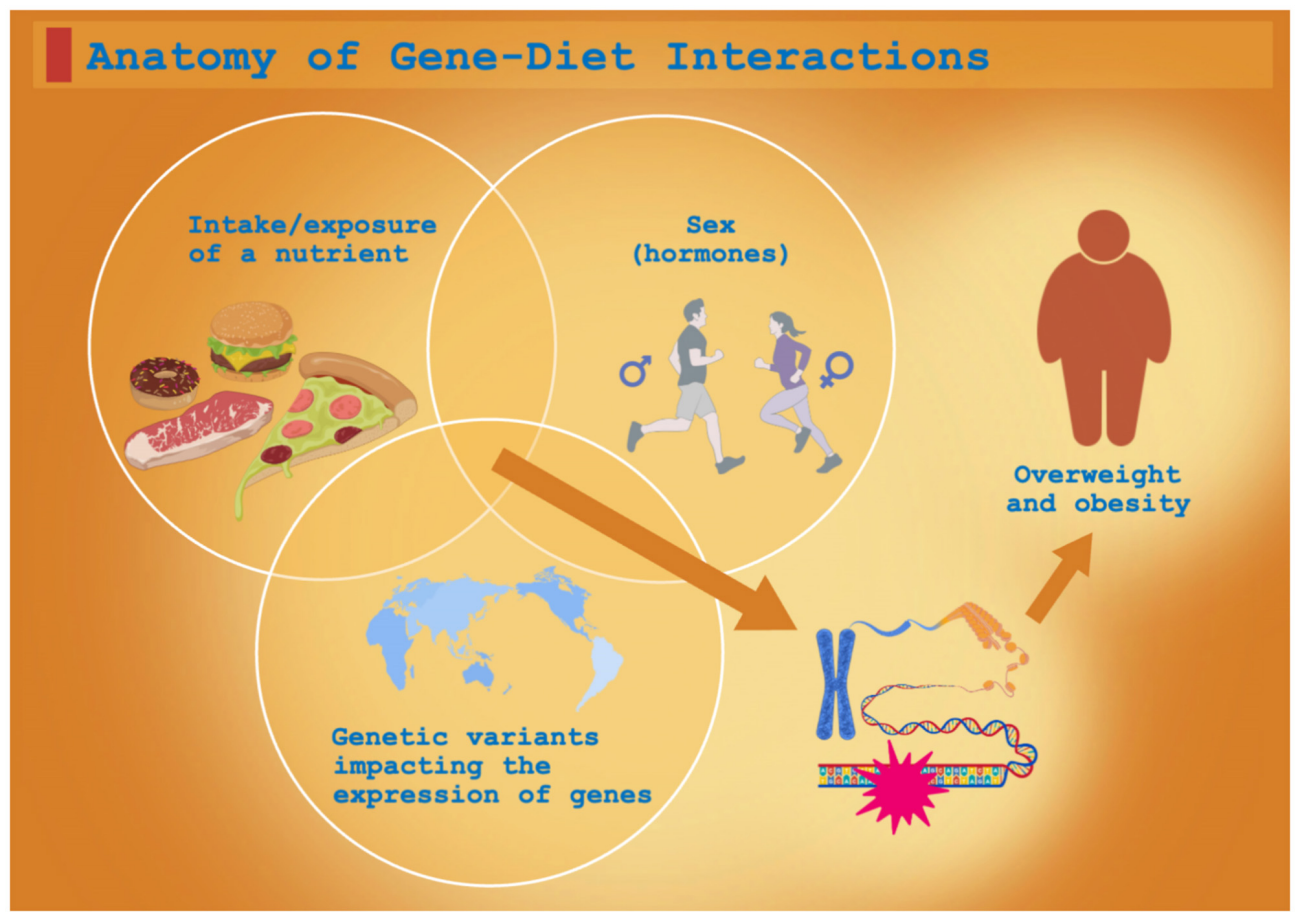
Anatomy of gene–diet interactions giving rise to clinical phenotypes.

This review synthesizes recent advances in understanding how dietary factors, specific SNPs, and their interactions influence obesity development, with the aim of informing the design of comprehensive interventions for overweight and obesity in Chinese adults (Figure 1).

## 2 Methods

This narrative review was conducted to synthesize and critically evaluate recent evidence on the interactions between SNPs, dietary patterns, and overweight/obesity in Chinese adults. The synthesis was structured around several key themes: the influence of dietary factors, the role of specific obesity-related SNPs (with attention to sex and ethnic differences), multigene synergistic effects, and the interplay between genetics and diet.

To elucidate this discussion, a literature search was performed in PubMed, China National Knowledge Infrastructure (CNKI), and Wanfang Data for studies published between January 2018 and April 2025. The literature search and study selection followed a structured process, as detailed in the Supplementary material. Given the substantial heterogeneity in study designs, exposures, and outcomes identified through this process, the evidence was integrated through a narrative synthesis, organizing the findings thematically to provide a coherent overview of the current research landscape.

## 3 Recent research on the influence of dietary factors on overweight and obesity in Chinese adults

Obesity is a major global public health challenge strongly influenced by multiple factors—with dietary macronutrients playing a key role. The overconsumption of dietary fat, specifically long-chain saturated fats, potently stimulates leptin secretion from expanding adipose tissue. Subsequently, circulating leptin crosses the blood–brain barrier to bind hypothalamic leptin receptors (*LepR*), primarily activating the *JAK2-STAT3* signaling pathway in *POMC* neurons. This activation ultimately promotes satiety and elevates energy expenditure, thereby facilitating the restoration of energy homeostasis (18, 19). However, chronic overnutrition induces a state of leptin resistance, which disrupts these regulatory pathways and leads to uncontrolled fat deposition (20). Longitudinal evidence shows a positive correlation between dietary fat intake and BMI (21). In contrast, findings from a prospective cohort study suggest that a dietary pattern with moderately high-fat content (30%−40% of energy) was associated with a lower risk of obesity (22). These apparent inconsistencies in macronutrient research highlight the constraints of adopting an isolated, nutrient-centric perspective. The metabolic response to dietary intake is significantly influenced by an individual's genetic makeup—a concept that will be further explored in the following sections. For example, polymorphisms in genes such as the fatty acid desaturase gene cluster 1 (*FADS1*) can modify fatty acid metabolism efficiency, making health outcomes dependent on both the specific nutrient and the individual's genotype (23–25). Similarly, variants in appetite-regulating genes including, the melanocortin 4 receptor (*MC4R*) and fat mass and obesity-associated gene (*FTO*), may enhance susceptibility to weight gain under high-energy diets (26, 27), whereas sex-specific genetic effects [e.g., in *ANK4* or ankyrin 1 (ANK1)] can lead to divergent dietary responses between males and females (28). Thus, the heterogeneity commonly observed in nutritional studies likely stems from the intricate interplay between dietary composition and genetic susceptibility, highlighting a critical dimension for future research. Although carbohydrates serve as the body's primary energy source, their relationship with obesity exhibits considerable complexity. Evidence indicates that low-carbohydrate diets may support weight management and enhance metabolic function (29). Notably, research reveals significant gender disparities in this association: higher carbohydrate intake is linked to a reduced risk of overweight and obesity among Chinese women, whereas this relationship remains less defined in men (30). Protein represents a pivotal component in macronutrient balance, with its effects being closely intertwined with the intake levels of carbohydrates and fats (22). Specifically, under free-living conditions, habitual high protein intake has been independently associated with elevated obesity risk (31). In contrast, within the context of specific weight-loss interventions such as ketogenic diets, increased protein consumption may support weight loss by enhancing satiety and promoting energy-expending processes including gluconeogenesis, which can incur an additional energy cost of 400–600 kcal per day (32). Similarly, the relationship between dietary fiber and obesity in Chinese adults exhibits a paradoxical complexity. Longitudinal studies demonstrate that neither total dietary fiber intake nor its intake from various food sources shows a significant association with obesity risk (33). In contrast, circulating levels of short-chain fatty acids (SCFAs)—such as butyrate derived from microbial fermentation of dietary fiber—are positively correlated with both BMI and central obesity (34). This paradox finds a critical explanation in intervention trials, which elucidate that dietary fiber (e.g., from fruits and vegetables) exerts significant benefits on weight reduction and metabolic health when it acts in concert with synbiotics that optimize gut microbiota composition (35).

The seemingly contradictory findings from single-nutrient studies collectively underscore the challenges in formulating consistent conclusions and emphasize the necessity of shifting research attention toward overall dietary patterns. Evidence suggests that specific macronutrient intake ratios (e.g., high-fat, low-carbohydrate patterns) are associated with a significantly elevated risk of obesity, whereas balanced energy distributions correlate with reduced risks of both obesity and related chronic diseases (36). Consequently, adopting a balanced dietary structure emerges as a fundamental strategy for effective obesity management and public health promotion.

The traditional Chinese diet, characterized by its plant-based composition, is increasingly being displaced by Western dietary patterns high in fat, sugar, and processed foods—a transition correlated with elevated obesity risk (37). Indeed, obesity incidence is twice as high among individuals consuming Western diets compared to those following traditional Chinese eating patterns (8). Regionally, traditional southern Chinese diets demonstrate protective effects against obesity (38–40), whereas northern diets—typically richer in refined carbohydrates and sodium—correlate with increased overweight risk, showing a 2.04-fold higher obesity prevalence than southern regions (38). Similarly, studies of Tibetan populations reveal that pastoral diets protect against central obesity (particularly in men), while urban diets high in red meat and refined carbohydrates elevate risks of metabolic syndrome and central adiposity (41, 42).

Dietary behaviors represent another critical dimension in obesity development. The practice of healthy eating habits—such as consuming light diets, selecting moderately textured foods, and eating slowly—has been shown to aid in the control and prevention of metabolic dysfunction (43). Notably, women and adults aged 45–60 years old demonstrate heightened susceptibility to unhealthy dietary practices (43). Beyond diet, other modifiable lifestyle factors, including smoking and alcohol consumption, constitute significant risk factors for abnormal BMI. Evidence indicates that smoking exhibits a dual association between underweight and overweight/obesity, whereas alcohol intake specifically elevates the risk of overweight and obesity. Collectively, these findings emphasize the multifactorial nature of lifestyle influences on BMI and underscore the importance of implementing integrated interventions that concurrently address these interrelated risk factors (44).

## 4 Recent progress on SNPs related to overweight and obesity in Chinese adults

**Figure 2:**
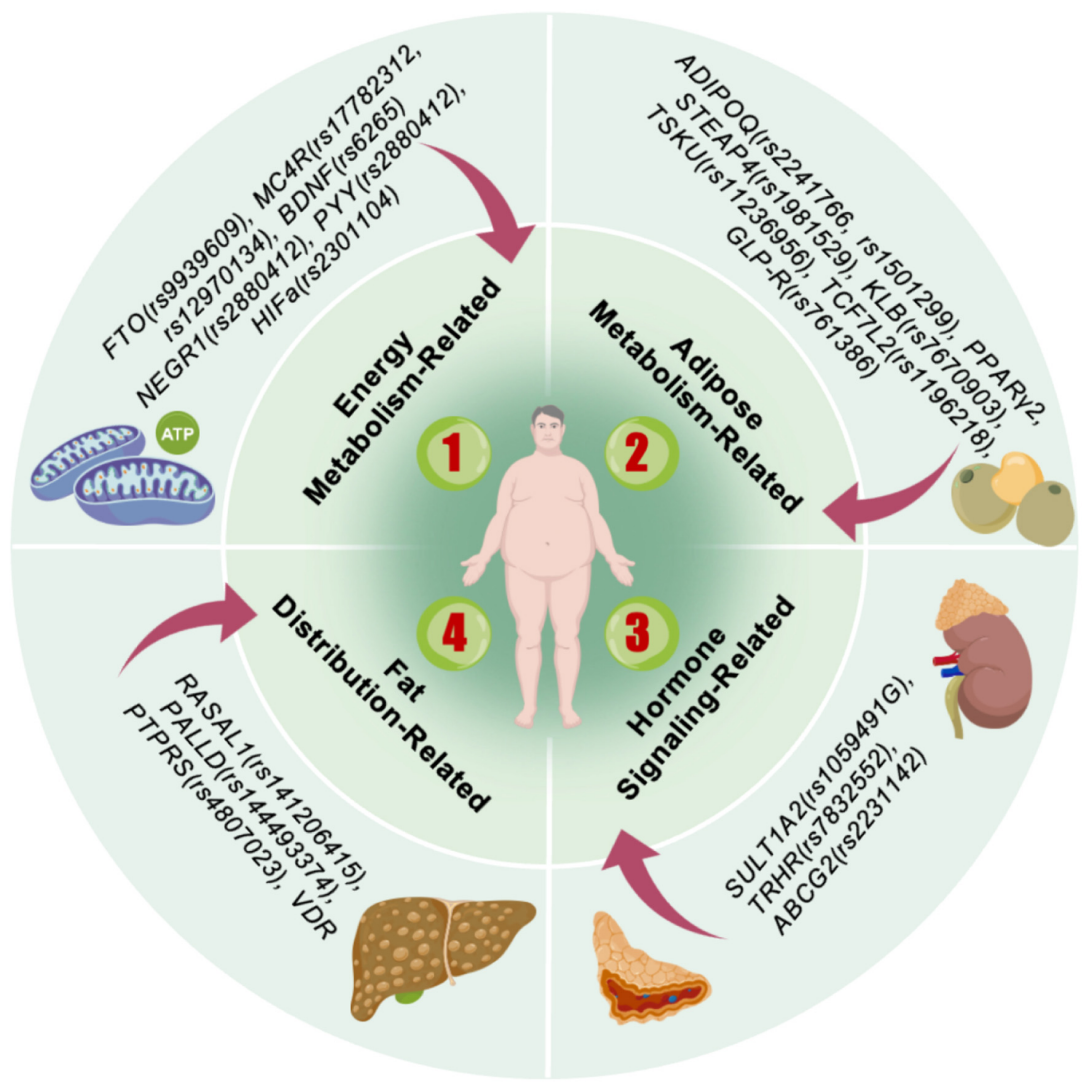
Main genes associated with obesity.

Obesity, commonly termed polygenic obesity, represents a quantitative trait arising from variations at multiple SNP loci. Collectively, these modest-effect variants are estimated to account for only approximately 5% of the observed heritability in obesity (14). Nevertheless, recent genome-wide investigations have revealed that obesity-associated SNP loci consistently demonstrate specific biological mechanisms. These include modulation of appetite regulatory pathways—exemplified by genes encoding brain-derived neurotrophic factor (*BDNF*) and *MC4R*—as well as genes participating in insulin secretion, energy metabolism, and lipogenesis, such as *FTO*, transcription factor 7-like 2 (*TCF7L2*), insulin receptor substrate 1 (*IRS1*), and forkhead box O3 (*FOXO3*) (13). This section systematically examines the relationship between SNPs (Figure 2) and overweight/obesity in Chinese adults, with particular emphasis on underlying functional abnormalities, sex- and ethnicity-specific regulation, and the synergistic effects exerted by multiple genes at key loci.

### 4.1 Function and regulation of core SNP loci

#### 4.1.1 Energy metabolism-related genes

The *FTO* gene, a well-established locus in obesity research, harbors SNPs (e.g., rs9939609) that elevate obesity susceptibility by modulating hunger and appetite pathways or by disrupting inter-locus balance (26, 45). This gene is associated with severe obesity in the Han Chinese population (46). Notably, the rs1121980 and rs17817449 loci showed no significant association with obesity in Tibetan cohorts, indicating ethnic-specific genetic effects (47).

The *MC4R* gene encodes a receptor pivotal in appetite and energy balance regulation. Its polymorphisms (e.g., rs17782313 and rs12970134) are linked to obesity susceptibility across diverse ethnic groups, including Han Chinese and Tibetan populations, demonstrating cross-ethnic relevance (26, 27, 47–49).

Gong et al. demonstrated that the *BDNF* polymorphism (rs6265) was correlated with BMI and waist circumference (WC) in sedentary individuals, likely mediated through its indirect role in energy balance via neuroplasticity (26). Liu et al. reported that the peptide YY (*PYY)* gene polymorphism (rs2880412) displayed a dual effect on obesity susceptibility: the CC genotype elevated obesity risk, whereas the AA genotype conferred protection through the modulation of lipoprotein metabolism [triglycerides (TG)↑ and low-density lipoprotein cholesterol (LDL-C)↓] (50, 51).

Hypoxia-inducible factor 1 alpha (*HIF1*α), a key transcription factor mediating hypoxic responses and metabolic reprogramming in tumor cells (52), plays a critical role in lipid metabolism—promoting fatty acid synthesis, inhibiting lipolysis and fatty acid oxidation, and enhancing cholesterol metabolism—beyond its established functions in glucose metabolism and angiogenesis. Metabolic reprogramming enables cellular adaptation to physiological or pathological stressors, including hypoxia, nutrient overload, and inflammation (53). *HIF1*α activation disrupts the balance between glycolysis and fatty acid oxidation, promoting a “compensation-inhibitory model” wherein depleted core metabolic functions are compensated by other genes while non-essential pathways are suppressed (54), thereby exacerbating obesity. In contrast, evidence suggests that the rs2301104 CC/CG genotype may serve as a protective factor against obesity in Chinese adults by reducing pro-inflammatory mediators (55), indicating that the C allele may counteract obesity through modulations in lipid metabolism, energy expenditure, and inflammatory responses. Additionally, ferroptosis signaling has been proposed to limit *HIF1*α availability, thereby restraining adipose tissue expansion during obesity and alleviating associated metabolic dysregulation (56).

#### 4.1.2 Adipose metabolism-related genes

SNPs in the adiponectin, C1q, and collagen domain-containing (*ADIPOQ*) gene (rs2241766, rs1501299) are associated with reduced susceptibility to multiple obesity phenotypes, underscoring the importance of their encoded lipocalins in insulin sensitivity and inflammatory regulation (57). The Pro/Ala polymorphism in the peroxisome proliferator-activated receptor gamma 2 (*PPAR*γ*2*) gene shows geographical stratification, with carriers in southern China displaying elevated obesity risk (58). This pattern may reflect temperature-mediated selection pressure influencing gene expression (59). In the Xinjiang Uyghur population, Zhu et al. (60) reported a significant protective effect for the *STEAP4* rs1981529 CC genotype, although its expression decreased during adipocyte differentiation and maturation, indicating a primary role in early-stage obesity development. The klotho beta (*KLB*) gene encodes Klotho β, an essential co-receptor in the FGF21–*KLB*–FGFR signaling pathway. Its rs7670903 polymorphism is associated with increased BMI and participates in a cross-regulatory network with non-alcoholic fatty liver disease (NAFLD) pathological processes (61). Emerging genetic targets include the *TSKU* gene rs11236956-G allele, which has been linked to obesity-related metabolic disorders. Carriers of this allele exhibit elevated serum Tsukushi protein levels, suggesting its potential involvement in obesity development (62). The *TCF7L2* and glucagon-like peptide-1 receptor (*GLP1-R*) genes encode proteins involved in glucose homeostasis and glucose-stimulated insulin secretion. Studies have demonstrated that individuals with the AA/AG genotype of *TCF7L2* rs11196218 have a 2.54-fold higher obesity risk compared to GG genotype carriers. Similarly, the *GLP1-R* rs761386 T allele is recognized as a risk factor for obesity (63).

#### 4.1.3 Hormone signaling-related genes

Carriers of the rs1059491G allele in the sulfotransferase family 1A member 2 (*SULT1A2*) gene exhibited a 54% reduction in overweight/obesity risk compared to non-carriers, a phenomenon potentially mediated through its location within the *PPAR*γ*2* and *RXRA* transcription factor binding sites (64). Although these associations did not survive multiple testing corrections, they provide novel insights into the potential involvement of metabolic detoxification pathways in obesity regulation (65). Gong et al. demonstrated that the rs7832552 polymorphism in the thyrotropin-releasing hormone receptor (*TRHR*) gene independently influenced waist circumference variation through the thyroid hormone signaling axis, suggesting the existence of specific endocrine regulatory nodes for central obesity (26). Additionally, the Q141K missense mutation (rs2231142) in the ATP-binding cassette subfamily G member 2 (*ABCG2*) gene shows association with overweight/obesity independent of *FTO* effects. The underlying mechanism, possibly involving cross-regulation between uric acid (UA) metabolism and energy homeostasis, requires further investigation (66).

#### 4.1.4 Fat distribution-related genes

A genome-wide association study (GWAS) conducted in a Chinese Han population identified significant associations between the RAS protein activator-like 1 (*RASAL1*) gene (rs141206415) and the palladin (*PALLD*) gene (rs144493374) with trunk fat mass, whereas the protein tyrosine phosphatase receptor type S (*PTPRS*) gene (rs4807023) was significantly correlated with leg fat mass (64). Additionally, Shen et al. reported that the C allele at the Fok1 locus and the T allele at the Apal locus of the vitamin D receptor (*VDR*) gene were associated with elevated body fat percentage (BFP). Furthermore, the rs2239179 G allele and the Apal locus T allele were linked to increased triceps skinfold thickness, independently of BMI and waist circumference (67). These findings collectively suggest that genetic regulatory pathways for distinct adipose tissue depots may operate through independent mechanisms.

### 4.2 Sex differences in SNP associations between overweight and obesity in Chinese adults

SNPs associated with overweight and obesity in Chinese adults exhibit pronounced **sex** differences. For instance, the *LGR4* rs11029986 polymorphism significantly influences BMI through the regulation of adipose transformation in females while exerting a more limited effect in males (68, 69). Both *BMAL1* rs6486121 and *CYP19A1* rs752760 contribute to sexually dimorphic obesity phenotypes, potentially mediated by sex hormone-regulated variations in fat distribution (70–72). Moreover, SNPs in mitochondrial genes also demonstrate sex-specific associations: the *MT-ND6* 14431T>C mutation correlates with obesity in males, whereas the *MT-TF* 152T>C mutation elevates obesity risk in females (73). Similarly, the *MFN2* rs2295281 T allele and CT genotype are associated with increased overweight/obesity risk in males, while the TT genotype confers elevated risk in females (68). Furthermore, *SH2B1*—which regulates energy balance through insulin sensitivity and adipocyte function—shows male-specific obesity association at the rs7359397 locus (49).

### 4.3 The influence of multi-gene synergistic effects on overweight and obesity in Chinese adults

#### 4.3.1 Gene interactions and metabolic imbalance

Specific genotype combinations of *MC4R* rs17782313 and *SH2B1* rs7359397 (TC-CC and TC-CT) confer a substantially elevated obesity risk−15.58-fold higher than other genotypes—through synergistic regulation of the leptin–melanocortin pathway (49). Separately, polymorphisms in the estrogen receptor 1 (*ESR1*) gene (rs712221 TT) and peroxisome proliferator-activated receptor delta (*PPARD*) gene (rs2016520 TC/TT) impair estrogen receptor activity and fatty acid oxidation capacity, thereby reducing overall lipid metabolic efficiency. Consequently, even under controlled energy intake, obesity risk increases substantially, indicating that impairments in metabolic efficiency—mediated through disrupted energy balance—represent a central mechanism in obesity pathogenesis (69).

#### 4.3.2 Multigene risk assessment

Polygenic risk score (PRS) enhances obesity risk prediction and facilitates personalized weight management strategies by integrating genome-wide SNPs associated with obesity phenotypes through weighted summation based on GWAS effect sizes (74). This approach demonstrates superior predictive capability compared to single-SNP analyses. For instance, studies integrating *FTO* variants (rs9930501, rs9930506, and rs9932754) and *ADRB2* polymorphisms (rs1042713 and rs1042714) through PRS have successfully predicted obesity susceptibility in Malay–Chinese populations. Notably, when combined with randomized controlled trial (RCT) data, these studies revealed more substantial improvements in inflammatory markers following dietary interventions in high-PRS subgroups (75). However, as a statistical model, PRS cannot elucidate the biological mechanisms through which SNPs influence obesity or intervention responses, and its predictive accuracy may diminish when applied to populations of different genetic backgrounds.

The weighted gene score (WGS) model quantifies genetic risk by calculating the weighted sum of risk alleles across multiple SNP loci, normalized by the total weight. This approach accounts for the differential contribution of variants at distinct loci to disease susceptibility (76). For instance, studies investigating olfactory pathway genes—including olfactory receptor 2A2 (*OR2AK2*) and olfactory receptor 2L8 (*OR2L8*)—have revealed their roles in modulating food cue perception, energy allocation, and adipose storage. While individual SNPs within this pathway exhibited both positive and negative correlations with obesity risk, the aggregate WGS of all olfactory-related SNPs demonstrated a significant inverse association with obesity susceptibility (77). It should be noted, however, that the WGS in this study was computed under an additive genetic model, which may underestimate the contribution of SNPs operating through dominance or epistatic effects, potentially introducing bias if key functional variants are overlooked. In a related direction, WGS analysis of G protein-coupled receptor (GPCR) signaling pathway genes has identified specific genotypes linked to obesity risk, providing novel perspectives on the neuroendocrine regulation of energy homeostasis (78).

The generalized multifactor dimensionality reduction (GMDR) method, which accommodates discrete and quantitative covariates, is suitable for analyzing both dichotomous and continuous phenotypes across diverse population-based study designs (79). This approach demonstrates superior cross-validation consistency (CVC) and balanced testing accuracy. For instance, studies using GMDR have identified specific locus combinations—FokI, rs2239179, and Apal—associated with elevated body fat percentage (BFP) susceptibility, while a four-locus combination (FokI, rs2239179, Apal, and rs218980) has been linked to increased triceps skinfold thickness risk (67). However, as GMDR primarily focuses on detecting interactions, it may overlook the main effects of individual SNPs. Therefore, combining GMDR with single-locus analyses (e.g., linear regression) is recommended to obtain more comprehensive insights. Additionally, the computational burden increases exponentially with the number of interaction sites analyzed, which may compromise analysis efficiency, particularly when handling large-scale genomic datasets.

This study transcends conventional single-gene analytical frameworks by underscoring the crucial role of gene–gene interactions in obesity pathogenesis and establishing a predictive model through integration of multi-genomic data, thereby informing the development of precision nutrition strategies.

In summary, investigation of polygenic synergistic effects and their corresponding statistical approaches (e.g., PRS, WGS, and GMDR) has substantially enhanced our understanding of obesity's genetic architecture, overcoming limitations inherent in single-locus analyses. To systematically summarize the core obesity-associated genes addressed in this section and their functional attributes, Table 1 presents key information on these major loci—including biological functions, risk alleles, and related phenotypes. This table organizes principal genetic factors modulating energy homeostasis, adipocyte metabolism, hormonal signaling, and fat distribution in Chinese adults, thereby providing a structured genetic framework for subsequent exploration of interactions between these genetic backgrounds and dietary patterns.

**Table 1:** Summary of gene polymorphisms associated with obesity and metabolic phenotypes in Chinese populations.

**Gene (symbol)**	**Biological function**	**Polymorphism (rs ID)**	**Variant/consequence**	**Risk allele**	**Associated phenotype(s)**	**Key population(s)**	**Reference(s)**
*FTO*	Regulates hunger- and appetite-related pathways, energy balance, and body weight homeostasis; may affect adipocyte development and mitochondrial thermogenesis	rs9939609 rs1121980 rs17817449	rs9939609: T/A rs1121980: G/A rs17817449: T/G	rs9939609: A rs1121980: A rs17817449: G	Obesity (BMI ≥28.0 kg/m^2^) Morbid obesity (BMI ≥35.0 kg/m^2^) Overweight (24.0 ≤ BMI < 28.0 kg/m^2^)	Han Chinese population Tibetan population	(26, 45, 46)
*MC4R*	Mainly expressed in the hypothalamus; plays a central role in regulating appetite, energy balance, body weight, and energy homeostasis	rs17782313 rs12970134	rs17782313: T>C rs12970134: G>A	rs17782313: C rs12970134: A	Obesity (BMI ≥ 28 kg/m^2^) overweight (24 ≤ BMI < 28 kg/m^2^)	Tibetan adults (Qinghai, Tibet) Yi people (Sichuan) Maonan people (Guangxi) Han Chinese	(26, 27, 47–49)
*BDNF*	Involved in neuroplasticity; indirectly regulates energy balance, thereby affecting body weight-related phenotypes (e.g., BM, and waist circumference)	rs6265	T>C	C	Obesity (BMI ≥ 28.0 kg/m^2^) Central obesity (WC ≥ 90 cm for males and WC ≥ 85 cm for females)	Chinese adults born in the early 1960s (from the 2010–2012 China `Nutrition and Health Surveillance, CNHS, covering 31 provinces of China)	(26)
*PYY*	An appetite-regulating hormone secreted by L cells in the distal gastrointestinal mucosa; regulates food intake and is closely associated with obesity; gene located at 17q21.1, containing 4 exons and 3 introns	rs2880412	A/C	C	Obesity (BMI ≥ 28 kg/m^2^) Altered lipoprotein metabolism [AA genotype: increased triglycerides (TGs), decreased low-density lipoprotein cholesterol (LDL-C)]	Han Chinese adults (rural areas in Central China, aged ≥ 18 years)	(50, 51)
*HIF1α*	Regulates hypoxia response as a key transcription factor; modulates obesity-related inflammation and lipid metabolism; influences energy expenditure to affect obesity susceptibility	rs2301104	G/C	G	Obesity ( BMI ≥ 28 kg/m^2^); altered levels of pro-inflammatory cytokines (IL-6, TNF-α, IL-1β, and IL-8)	Han Chinese adults (160 obese cases and 166 age-/sex-matched healthy controls; excluded subjects with autoimmune/inflammatory diseases or taking metabolic drugs)	(55)
*PPARγ2*	Acts as a nuclear receptor, highly expressed in adipose tissue; regulates adipogenesis (fat cell formation) and energy metabolism; Involves in lipid metabolism and insulin sensitivity; Its Pro12Ala polymorphism affects transcriptional activity and adipogenic potential	Pro12Ala	CCA → GCA mutation at codon 12 of *PPARγ2* gene exon; Pro/Pro: wild-type genotype; Pro/Ala: heterozygous mutant genotype; - Ala/Ala: homozygous mutant genotype	Ala	Obesity (BMI ≥ 28 kg/m^2^)	Chinese adults (covering seven provinces including Sichuan, Hebei, Shaanxi, Shandong, Liaoning, Ningxia, and Jiangxi); stratified into southern and northern regions (with stronger correlation in southern regions)	(58)
*STEAP4*	Highly expressed in human preadipocytes, with gradually downregulated expression as adipocytes differentiate and mature; Promotes adipocyte differentiation, maturation, and lipid accumulation; Located on chromosome 7q21.12 (a susceptibility region for type 2 diabetes and insulin resistance); may be involved in the early stage of obesity development	rs1981529	Located in exon 2 of the *STEAP4* gene; genotypes: TT, TC, and CC; alleles: T and C; CC genotype frequency: 4.4% in the non-obese group vs. 2.0% in the overweight/obese group	T	Overweight and obesity (BMI ≥ 25 kg/m^2^)	Uygur adults in Hetian, Xinjiang (30–70 years old); excluding subjects with secondary obesity, tumors, or severe liver/kidney diseases; total of 2,048 subjects (893 non-obese and 1,155 overweight/obese)	(60)
*KLB*	A key co-receptor in the *FGF21-KLB-FGFR* signaling pathway; mediates the binding of FGF21 to FGFR to activate intracellular signaling; regulates energy homeostasis and metabolic processes (especially in liver and adipose tissue); involved in the cross-regulatory network with non-alcoholic fatty liver disease (NAFLD) pathological processes	rs7670903	Located in intron 3 of *KLB*, near the end of exon 3 in the protein coding domain; alleles: A (minor allele), G (major allele); A allele correlates with higher BMI (*p* = 0.0005)	A	Obesity (BMI ≥25 kg/m^2^, Asian population standard)	Unrelated Han Chinese adults (18–80 years old) from the Physical Examination Center of the Affiliated Hospital of Xuzhou Medical University	(61)
*TSKU*	A secreted protein of the leucine-rich proteoglycan family; acts as a hepatokine involved in regulating energy balance and metabolic processes; associates with obesity severity and glucose homeostasis; influences visceral fat accumulation and insulin secretion	rs11236956	A/G	G	Obesity (defined as BMI ≥28 kg/m^2^); Obesity-related metabolic disorders: increased visceral fat area (VFA), elevated plasma glucose after the oral glucose tolerance test (OGTT), insulin resistance (reduced Stumvoll insulin secretion index), increased free fatty acids (FFA), elevated γ-glutamyltransferase (γ-GT)	Han Chinese adults from the Shanghai community (total *n* = 11,022; obese subgroup *n* = 18,79); excluded subjects with surgery, trauma, pregnancy, cancer, severe liver/kidney disease, or psychiatric disturbance	(62)
*TCF7L2*	Encodes a high-mobility group (HMG) box-containing transcription factor; Plays a key role in the Wnt signaling pathway; involved in glucose homeostasis; influences β-cell function and insulin secretion	rs11196218	A/G	A	Obesity (BMI ≥28 kg/m^2^, or male WC ≥90 cm/female WC ≥85 cm)	Chinese Han adults (aged ≥18 years) from Xiangya Hospital, Central South University; 60 obese cases (fasting blood sugar FBG < 5.6 mmol/L, no diabetes history); 69 non-obese controls (BMI 18.5–23.9 kg/m^2^); sex- and age–matched controls	(63)
*GLP1-R*	Encodes a 7-transmembrane protein; functions as a receptor for glucagon-like peptide 1 (*GLP-1*); stimulates glucose-induced insulin secretion; involved in glucose homeostasis	rs761386	C/T	T	Same as above	Same as above	Same as above
*SULT1A2*	Belongs to the sulfotransferase (SULT) superfamily; catalyzes sulfonation of phenolic compounds, participating in phase II metabolic detoxification; maintains endocrine homeostasis by metabolizing hormones (e.g., estrogens and 17β-estradiol); encodes heat-resistant diphenol sulfotransferase, a key enzyme in sulfuric acid metabolism; located at transcription factor binding sites of *PPARγ2* and *RXRA*, potentially regulating lipid metabolism and obesity	rs1059491	c.704 T/G (missense mutation, p.Asn235Thr); genotypes: TT (wild-type), GT (heterozygous), and GG (homozygous); minor allele frequency (MAF): 0.0292 in the overweight/obese group, 0.0686 in the normal weight group; located in exon 7 of the *SULT1A2* coding region	T	Overweight (25 ≤ BMI < 30 kg/m^2^); obesity (BMI ≥ 30 kg/m^2^); hypertriglyceridemia (fasting TG ≥ 1.7 mmol/L); Dyslipidemia (presence of TC ≥ 5.2 mmol/L, LDL-C ≥ 3.4 mmol/L, HDL-C < 1.0 mmol/L, or TG ≥ 1.7 mmol/L)	Southern Chinese adults (Han ethnicity) from Taizhou, Zhejiang; Total of 466 subjects (226 normal weight, 168 overweight, and 72 obese); excluded: pregnant/lactating individuals, those taking weight/energy metabolism-affecting drugs, patients with mental illness, gastrointestinal disorders, or morbid obesity	(65)
*ABCG2*	Encodes a high-capacity urate transporter; regulates renal and intestinal uric acid (UA) excretion; involves in cross-regulation of uric acid metabolism and energy homeostasis; its polymorphism affects lipid metabolism (triglyceride, HDL)	rs2231142 (Q141K)	Missense mutation: c.421G>T, leading to amino acid substitution Glu141Lys (Q141K); genotypes: GG (wild-type), GT (heterozygous), and TT (homozygous); alleles: G (major) and T (minor); minor allele frequency (MAF): T allele = 43.0% in the overweight/obesity group, 18.9% in the normal weight group	T	Overweight (24 ≤ BMI < 28 kg/m^2^); obesity (BMI ≥ 28 kg/m^2^); hyperuricemia (elevated serum UA levels); dyslipidemia [elevated triglycerides (TG), decreased high-density lipoprotein (HDL)]	Han Chinese adults from the Huangjiahu Hospital physical examination population; Total of 376 subjects (193 overweight/obese and 183 normal weight); stratified into total cohort (28–68 years), youth cohort (28–40 years), and middle-aged and elderly cohort (41–68 years); excluded: subjects with acute/chronic diseases, family genetic diseases, pregnancy/lactation, or taking metabolism-affecting drugs	(66)
*LGR4*	Regulates osteoblast differentiation and bone mass via the Wnt/β-catenin signaling pathway; involved in glycolipid metabolism and energy homeostasis; modulates adipose transformation (white-to-brown fat switch) to affect body weight control; its polymorphism shows gender-specific effects on BMI regulation	rs11029986	Located in intron 10 of the *LGR4* gene; alleles: C (major) and T (minor); in male cohorts: significantly associated with total fat mass (TFM) and percentage of TFM (PFM) (*p* = 0.014 and 0.011, respectively) after 1,000 permutations; in female cohorts: excluded due to minor allele frequency (MAF) < 0.05, showing weaker or no association with BMI-related phenotypes	Not explicitly identified	Gender-specific effect: significantly affects BMI by regulating adipose transformation in females, while the effect is weaker in males	Chinese Han male nuclear families (Document 1): 400 male-offspring nuclear families (1,215 individuals, including 339 young men aged 20–40 years); excluded: subjects with metabolic diseases (diabetes, hypertension, etc.), BMI >25 kg/m^2^, or taking osteoporosis-related drugs; -Chinese Han female nuclear families (Document 2): 379 female-offspring nuclear families (1,262 individuals, including 467 young women aged 34.1 ± 6.8 years); Excluded: Subjects with chronic diseases, endocrine disorders, or secondary low bone mass	(95, 96)

## 5 Recent progress of interactions between SNPs and diet on overweight and obesity in Chinese adults

While each gene locus exerts a modest effect on obesity, its interaction with dietary factors leads to complex phenotypic outcomes, thereby accounting for the observed variations in nutritional intake behaviors and energy metabolism among individuals with different genetic backgrounds (80).

### 5.1 The effects of specific dietary interventions in populations with different genotypes are modulated by multiple factors

High-energy diets are prevalent worldwide, and the interaction of *MC4R*—a key gene regulating appetite and energy balance—plays a critical role. Gong et al. conducted a 2-year prospective cohort study of 2,216 Chinese adults from the 2010–2012 China Nutrition and Health Survey. The researchers found that carriers of the *MC4R* rs12970134 risk allele experienced an average BMI increase of 0.140 kg/m^2^ (*p* = 0.049) when consuming a high-energy diet (defined as total energy intake above the median), compared to wild-type individuals (26). This gene–diet interaction was similarly observed in a Yi migrant population, where carriers of the rs17782313 C/C genotype exhibited a tenfold higher obesity risk following rural-to-urban migration (27), potentially linked to greater exposure to high-fat and high-sugar foods in urban diets. This mechanism further explains the persistent rise in obesity rates in modern Chinese society against a stable genetic background (81).

Further investigating gene–diet interactions, Gong et al. (26) reported that carriers of the *FTO* rs8050136 risk allele exhibited a 77% increased obesity risk when consuming a high-energy diet. The researchers also observed that *FTO* was positively correlated with dietary fat intake proportion and inversely correlated with carbohydrate energy share (82), suggesting that this gene influences obesity susceptibility partly through modulating dietary behaviors. This principle—that genetic background shapes dietary response—is further demonstrated in intervention trials. In an 8-week RCT involving Chinese overweight/obese adults (mean baseline BMI ~27 kg/m^2^), Kuang et al. (83) showed that the weight reduction efficacy of defatted flaxseed flour (13.5 g fiber/100 g) was strictly genotype-dependent. Significant body weight reductions occurred exclusively in carriers of specific alleles: the A allele of *FTO* rs11076023 (−2.48 kg), the T allele of *PCSK1* rs155971 (−2.81 kg), and the A allele of *BDNF* rs6265 (−2.34 kg), with no significant effect in non-carriers (*p* for interaction < 0.05 for all). These findings underscore that nutritional interventions can produce divergent effects—even promoting weight gain in certain genetic subgroups—thus challenging the universal application of dietary guidelines and emphasizing the crucial role of nutrigenetics in identifying potential responders (84).

As described previously, PRS further expands the scope of personalized nutrition. In a 6-month RCT involving 128 overweight/obese Malaysian adults (mean baseline BMI ~29.5 kg/m^2^), Tan and Mitra (75) examined interactions between a PRS—integrating *FTO* (rs9930501, rs9930506, and rs9932754) and *ADRB2* (rs1042713, rs1042714) variants—and a high-protein, energy-restricted, high-vitamin E, high-fiber (Hipcref) diet. Notably, participants in the highest PRS tertile (range: 3.60–8.18) achieved substantially greater weight loss (−4.6 kg) following the Hipcref intervention than those in the lowest tertile (−2.5 kg). Waist circumference reduction was also more pronounced in the high-PRS group (−7.2 vs. −5.2 cm). Moreover, the high-PRS group exhibited a significantly larger decrease in high-sensitivity C-reactive protein (hsCRP) under the Hipcref diet relative to the control diet, indicating an enhanced anti-inflammatory response among genetically predisposed individuals (75).

Nevertheless, the limitations of SNPs in predicting dietary intervention efficacy warrant consideration. Despite adherence to a high-fiber, low-energy diet, carriers of the *ESR1* rs712221 TT and *PPARD* rs2016520 TC/TT genotypes exhibited significantly greater increases in BMI and waist circumference than non-carriers (69). This effect may be explained by the *PPARD* rs2016520 TC/TT genotype impairing fatty acid oxidation, combined with the *ESR1* rs712221 TT genotype disrupting fat distribution regulation. This specific genotypic combination appears to strongly shape obesity predisposition by reprogramming metabolic priorities—an effect that appears resistant to dietary intervention. These findings challenge the generalizability of universal dietary recommendations and highlight the necessity of precision nutrition to identify genotype-imposed constraints on metabolic plasticity. Future research should aim to develop a three-dimensional response map integrating genotype, dietary composition, and metabolic phenotype to facilitate dynamic correction of compensatory metabolic defects.

### 5.2 Genetic polymorphisms in nutrient metabolism genes modulate dietary responses

In addition to direct regulation, SNPs may also indirectly influence metabolic networks, leading to differences in dietary responses associated with polymorphisms in nutrient metabolic pathways. Metabolic flux redistribution—a process wherein metabolite flow is altered in metabolic networks due to genetic variation, environmental changes, or intervention—can significantly impact cellular physiology. Enzymes encoded by *FADS1*/2 serve as central catalysts in n-3/n-6 polyunsaturated fatty acid (PUFA) metabolism. Rs174547 TT genotype carriers displayed reduced high-density lipoprotein cholesterol (HDL-C) levels [(1.1 ± 0.3) mmol/L vs. (1.4 ± 0.3) mmol/L] under moderate-to-high linoleic acid (LA) intake (23). This likely stems from the TT genotype impairing *FADS1* catalytic efficiency in converting alpha-linolenic acid (ALA) to eicosapentaenoic acid/ docosahexaenoic acid (EPA/DHA; conversion rate only 5%−10%) (24), while high LA further competitively inhibits the n-3 pathway, creating a metabolic bottleneck. Consequently, ALA supplementation failed to reverse the TT phenotype [HDL-c: (1.2 ± 0.3) mmol/L vs. (1.3 ± 0.5) mmol/L, *p* > 0.05] (25). These findings underscore the need to integrate multi-omics data in future studies to elucidate how key SNPs modulate metabolic flux redistribution and influence obesity, thereby establishing a cross-scale theoretical framework for precision nutrition.

Notably, although n-3 PUFAs are generally considered beneficial for weight management and the rs174570 T allele has been identified as an adaptive marker linked to high-fat fish and n-3 PUFA intake, carriers of this allele showed greater BMI increases following high fish consumption (≥1 time/day) than non-carriers [BMI change: (1.55 ± 0.25) kg/m^2^ vs. (1.15 ± 0.13) kg/m^2^] (85). This paradoxical observation may result from linkage disequilibrium between rs174547 and rs174570 (86), which jointly alter desaturase activity and thereby transform conventionally beneficial dietary components into metabolic liabilities in specific genetic backgrounds.

The potent antioxidant properties of haptoglobin (Hp) offer additional insight into the metabolic benefits of dietary interventions (87). Under balanced dietary regimens, Hp 1-1 carriers with elevated free hemoglobin-binding capacity exhibited more substantial reductions in abdominal adiposity (waist circumference: −7.91 cm vs. −5.19 cm) and greater improvement in the homeostasis model assessment of insulin resistance (HOMA-IR) (88). These findings underscore the critical role of antioxidant activity in mitigating oxidative stress induced by high-fat diets. Nevertheless, the influence of the Hp phenotype is dually modulated by pro-inflammatory factors such as IL-6 and TNF-α, as well as nutritional status (89, 90), indicating that future interventions should integrate anti-inflammatory dietary strategies to enhance efficacy.

Research on angiotensin-related genes (e.g., *AGTR1*/2) further demonstrates that healthy dietary patterns can partially counteract genetically driven adverse lipid profiles through genotype-specific mechanisms. Specifically, only carriers of the AGTR rs1403543 AA genotype exhibited U-shaped distributions of total cholesterol (TC), LDL-C, and HDL-C under the vegetable–fruit–soybean (VFSD) dietary pattern (91), with no significant associations observed for other genotypes or dietary patterns such as REFD. In a similar vein, the small ubiquitin-like modifier 4 (*SUMO4*) gene rs237025 polymorphism causes an M55V substitution that enhances nuclear factor kappa B (NF-κB) expression, promoting insulin resistance and lipid accumulation in adipocytes. Conversely, Shandong participants carrying the GG genotype showed improved lipid metabolic profiles when adhering to weight management strategies involving dietary modification and salt restriction (92).

### 5.3 Sex-related gene–diet interaction

**Sex** differences not only influence SNPs, as discussed in the previous section, but also play a significant role in gene polymorphism–diet interactions.

Notably, the rs516946 T allele of the ankyrin 1 (*ANK1*) gene significantly counteracted adverse dietary iron effects in men, but not in women (28). This sex-specific effect may be attributed to the absence of cyclic blood loss in males, which enables the *ANK1* genotype to mitigate oxidative damage-related metabolic disorders by enhancing iron metabolic efficiency. Furthermore, Zhao et al. demonstrated that rs429358 exerts stronger effects in males by regulating apolipoprotein E (*APOE*) production and influencing lipid metabolic pathways, representing a key determinant of lipid profiles (93). Their findings further indicated that maintaining ideal body composition in men requires emphasis on high-quality protein sources, with eggs being preferable to red meat (93).

Multiple SNPs are associated with female body shape regulation (Table 2) (93), revealing a complex network of gene–nutrition interactions. For instance, rs4994 regulates *ADRB3* protein expression—a key mediator of lipolysis and energy reserve modulation—thereby contributing to individual variations in energy expenditure, with more pronounced effects in females (93). Substantial heterogeneity exists in the metabolic responses to nutrient sources among women with different genotypes. Carriers of either the ANKK1 rs1800497 GG/GA or *FABP2* rs3751812 GG genotype, for example, exhibit synergistic benefits for body size maintenance when consuming nuts and other high-quality fat sources. Carbohydrate responsiveness also varies genotypically: the rs1800497 AA and rs1800544 GG genotypes show particularly distinct responses (93). The AA genotype reduces dopamine receptor D2 (*DRD2*) density, promoting increased sugar consumption for neural reward stimulation and thereby elevating obesity risk (93). In contrast, the G allele elevates fasting glucose levels in Chinese individuals, predisposing them to central obesity. Macronutrient source specificity is further evidenced by the rs662799 polymorphism, wherein AG and AA genotypes respond differentially to plant vs. animal proteins (93). Interestingly, fried food consumption does not appear to adversely affect body shape among AG-genotype women, a subgroup well-represented in the Chinese Han population (93).

**Table 2:** Information on important SNPs with high impact on Chinese women (93).

**Phenotype category**	**Gene**	**RS-ID**	**REF**	**ALT**	**Ref**	**Alt**
Body mass composition related	*APOA5*	rs662799	A	G	0.73	0.27
	*ANKK1*	rs1800497	G	A	0.57	0.43
Size dimension related	*APOE*	rs429358	T	C	0.85	0.15
	*FTO*	rs3751812	G	T	0.87	0.13
	*MC4R*	rs17782313	T	C	0.77	0.23
	*ADRA2A*	rs1800544	G	C	0.7	0.3

## 6 Conclusion and future perspective

Obesity, as a complex polygenic disease, arises from the interplay of genetic susceptibility, environmental factors (particularly dietary patterns), and their interactions. This review has synthesized recent advances in obesity-related genetic SNPs and gene–diet interactions, highlighting the crucial roles of multigene synergism, metabolic network regulation, and sex differences in obesity pathogenesis.

Despite notable progress, current evidence remains largely static, failing to elucidate the critical timing and mechanisms behind the “triggering event” in the “genes load the gun, environment pulls the trigger” paradigm. Furthermore, the majority of studies are constrained by limited sample sizes and brief intervention durations, hindering the assessment of long-term exposure cumulative effects and metabolic compensation sustainability. The scarcity of cross-regional validation also restricts the generalizability of reported associations.

Looking forward, it is imperative to construct a three-dimensional gene–diet–metabolic phenotype model via long-term, large-scale multi-ethnic cohort studies to identify critical windows and reversible mechanisms of metabolic dysregulation. This approach will accelerate the transition from universal dietary recommendations to precision nutrition. Furthermore, integrating artificial intelligence and machine learning can enable dynamic tools to quantify non-linear gene–diet interactions and metabolic flux redistribution patterns. For instance, a recent BMI prediction model leveraging gut microbiome SNP density and random forest algorithms demonstrates enhanced efficacy (94). Future studies should also integrate genomic, metabolomic, and immunomic data to elucidate how multi-level regulatory networks shape obesity phenotypes. Particular attention should be paid to the unique characteristics of Chinese populations, with more localized research initiatives needed to inform region-specific prevention and control strategies.
