# Supplementary material for: Research progress on the mechanistic impact of single-nucleotide polymorphisms and dietary pattern interactions on overweight and obesity in Chinese adults: a narrative review
Source: Front Nutr. 2025 Nov 18;12:1603038. doi: 10.3389/fnut.2025.1603038 (PMC12668921; doi:10.3389/fnut.2025.1603038)
Supplement: Supplementary file 1 [file Table_1.docx]

***Supplementary Material***

**Supplementary Methods**

**S1. Detailed Search Strategy**

The systematic literature search was conducted using the following specific search strings in each database. The strategy was constructed using an iterative combination of keywords and Medical Subject Headings (MeSH) terms.

**PubMed:**

1. ("Macronutrient" OR "Fat intake" OR "Carbohydrate intake" OR "Protein intake" OR "Dietary Behavior " OR "Dietary Fiber" OR "Dietary pattern") AND ("obesity" OR "overweight") AND ("Chinese adult" OR "Chinese")
2. ("SNPs" OR "Genetic polymorphism") AND ("obesity" OR "overweight") AND ("Chinese adult" OR "Chinese")
3. ("PRS" OR "GWAS") AND “obesity” OR “overweight” AND ("Chinese adult" OR "Chinese")
4. ("Gene-diet interactions" OR "gene-environment interactions") AND “obesity” OR “overweight” AND ("Chinese adults" OR "Chinese")

Corresponding search strategies were translated and adapted for the Chinese databases (CNKI and Wanfang Data). No language restrictions were applied. Furthermore, an additional backward-and-forward citation search was performed on the included studies.

**S2. Study Selection Process**

A PRISMA-style flow diagram illustrating the study selection process is provided below (**Supplementary Figure S1**).

**Identification of studies via databases and registers**

Records identified from*:

Databases (n = 6662)

Duplicate records removed (n = 5822)

**Identification**

Records screened

(n = 840)

Records excluded

(n = 344)

**Screening**

Reports excluded:

- Reviews, editorials, books and book chapters, notesletters, conference papers, surveys (n = 112)
- In vitro or animal studie (n = 46)
- Monogenic and/or syndromic obesity (n = 2)
- Investigation for other mutations except SNPs or CNVs (n = 6)
- Pharmacological or bariatric surgery obesitymanagement interventions (n = 66)
- Other outcomes examined and not BMI and/or bodycomposition change (e.g., gene expression) (n = 214)

Reports assessed for eligibility

(n =496)

Studies included in review

(n = 50)

**Included**

*Supplementary Figure S1.* Flowchart of the study identification and selection process.

**S3. Detailed Eligibility Criteria**

**Inclusion Criteria:**

- Age≥ 18 years
- Any language
- China
- First published between January 2018 and April 2025
- Species: Humans
- BMI: overweight/obesity
- Common/polygenic obesity
- Qutcomes examined: change in BMland/or body composition inrelation to the genotypeExamining the effect of SNPs

**Exclusion Criteria:**

- Age＜ 18 years
- In vitro or animal studies
- Reviews, editorials, books and book chapters, notesletters, conference papers, surveys
- Preventive intervention programs forobesity development
- Monogenic and/or syndromic obesity
- Investigation for other mutations except SNPs or CNVs
- Studies examining BMI and/or body composition changeat a time after the end of a lifestyle intervention program
- Pharmacological or bariatric surgery obesitymanagement interventions
- Other outcomes examined and not BMI and/or bodycomposition change (e.g., gene expression)

**S4. Data Extraction Items**

Data from each included study were extracted into a standardized form, capturing the following:

- Study characteristics: First author, publication year, study design (e.g., cross-sectional, cohort, RCT), location in China, sample size.
- Population details: Mean age and standard deviation, sex distribution (%), specific ethnic subgroup (e.g., Han, Tibetan, Uyghur) if reported.
- Genetic factors: Gene symbol(s), specific SNP identifier(s) (rs number), genotyping method, risk allele frequency or genotype distribution.
- Dietary assessment: Method of dietary intake assessment (e.g., Food Frequency Questionnaire (FFQ), 24-hour recall, dietary history), specific dietary exposures (e.g., dietary pattern scores, nutrient intake levels, food groups).
- Key findings: Primary obesity outcomes and measures of association (e.g., β-coefficients with confidence intervals, odds ratios with confidence intervals, p-values). For interaction analyses, the statistical model and p-value for the interaction term were extracted. All sex-stratified results were specifically noted.
